# Structure of recombinant *Leishmania donovani* pteridine reductase reveals a disordered active site

**DOI:** 10.1107/S174430911004724X

**Published:** 2010-12-21

**Authors:** Keri L. Barrack, Lindsay B. Tulloch, Lynsey-Ann Burke, Paul K. Fyfe, William N. Hunter

**Affiliations:** a Division of Biological Chemistry and Drug Discovery, College of Life Sciences, University of Dundee, Dundee DD1 5EH, Scotland

**Keywords:** antifolates, pteridine reductase, *Leishmania*, pterins, *Trypanosoma*

## Abstract

Pteridine reductase (PTR1) is a potential target for drug development against parasitic *Trypanosoma* and *Leishmania* species, protozoa that are responsible for a range of serious diseases found in tropical and subtropical parts of the world. As part of a structure-based approach to inhibitor development, specifically targeting *Leishmania* species, well ordered crystals of *L. donovani* PTR1 were sought to support the characterization of complexes formed with inhibitors. An efficient system for recombinant protein production was prepared and the enzyme was purified and crystallized in an orthorhombic form with ammonium sulfate as the precipitant. Diffraction data were measured to 2.5 Å resolution and the structure was solved by molecular replacement. However, a sulfate occupies a phosphate-binding site used by NADPH and occludes cofactor binding. The nicotinamide moiety is a critical component of the active site and without it this part of the structure is disordered. The crystal form obtained under these conditions is therefore unsuitable for the characterization of inhibitor complexes.

## Abstract 2

The structure of *L. donovani* pteridine reductase has been targeted to assist in a program of structure-based inhibitor research. Crystals that diffracted to 2.5 Å resolution were obtained and the structure has been solved. Unfortunately, the active site is disordered and this crystal form is unsuitable for use in characterizing enzyme–ligand interactions.

## 1. Introduction

*Leishmania* are protozoan parasites (order Trypanosomatida, class Kinetoplastida) that cause a range of diseases and present a serious health risk to millions of people worldwide (Desjeux, 2004 ▶; Reithinger *et al.*, 2007 ▶). The incidence of infection is primarily in tropical and subtropical regions of the world (Herwaldt, 1999 ▶). The different species of *Leishmania* are responsible for distinctive conditions (Reithinger *et al.*, 2007 ▶). For example, *L. donovani* causes visceral leishmaniasis, a potentially fatal disease, while infection with *L. major* leads to cutaneous leishmaniasis. Several com­pounds are available to treat these infections, but increasing levels of drug resistance combined with the high cost and toxicity of anti­leishmanial drugs compromises the control of the diseases (Croft *et al.*, 2006 ▶; Maltezou, 2010 ▶). These observations explain in part why the World Health Organization has identified the leishmaniases as neglected diseases and is urgently seeking novel therapeutic approaches (World Health Organization, 2007 ▶).

Our aim is to identify and characterize drug targets in these parasites and to apply structure-based approaches to develop potent inhibitors that possess the right chemical properties to underpin early-stage drug discovery (Hunter, 2009 ▶). A promising target with respect to infection with *Leishmania* sp. is the NADPH-dependent short-chain dehydrogenase/reductase pteridine reductase (PTR1; EC 1.5.1.33). This enzyme is unique to trypanosomatid parasites, where it supports the provision of reduced biopterins that are necessary for meta­cyclogenesis (Cunningham *et al.*, 2001 ▶) and which are implicated in resistance to reactive oxygen and nitrogen species in *Leishmania* (Moreira *et al.*, 2009 ▶). PTR1 catalyzes the reduction of biopterin to 7,8-dihydrobiopterin as well as its subsequent reduction to 5,6,7,8-tetrahydrobiopterin. Additionally, the enzyme catalyzes similar reactions in the salvage of unconjugated folates in *Leishmania* (Nare, Hardy *et al.*, 1997 ▶; Nare, Luba et al., 1997 ▶). As *Leishmania* are auxotrophic for pteridines (folates and pterins) and are required to obtain these nutrients from their environment to maintain growth, disrupting this salvage process represents a potential therapeutic strategy.

We have previously studied the structure–mechanism–activity relationships for the enzymes from *L. major* (*Lm*PTR1) and *Tryp­ano­soma brucei* (*Tb*PTR1) and determined the structures of a series of inhibitor complexes (Gourley *et al.*, 2001 ▶; Dawson *et al.*, 2006 ▶; Cavazzuti *et al.*, 2008 ▶; Mpamhanga *et al.*, 2009 ▶), whilst the structure of *T. cruzi* PTR2 has been reported by others (Schormann *et al.*, 2005 ▶). We identified sequence and structural differences between *Lm*PTR1 and *Tb*PTR1 that explain why some inhibitors display a significant level of selectivity for one orthologue over the other (Gibson *et al.*, 2009 ▶; Tulloch *et al.*, 2010 ▶). Although we were able to routinely generate crystals of *Tb*PTR1 that diffracted to between 2.0 and 1.0 Å resolution (Dawson *et al.*, 2010 ▶), studies with *Lm*PTR1 have been hampered by poor crystal quality and a lack of reproducibility. One crystal form of *Lm*PTR1 diffracted to beyond 2.0 Å resolution but could only be obtained in the presence of NADPH and methotrexate (Gourley *et al.*, 2001 ▶); when other ligands were present different crystal forms were obtained. The size of the asymmetric unit is increased from two to either four or eight subunits and the crystals are often mechanically twinned and diffract to lower resolution, with the diffraction pattern being anisotropic and highly mosaic (McLuskey *et al.*, 2004 ▶; Schüttelkopf *et al.*, 2005 ▶). An alternative source of *Leishmania* PTR1 was therefore sought for our investigations. Studies with *L. tarentolae* PTR1 resulted in a 2.8 Å resolution structure of the complex with NADPH, but despite its presence in the crystallization mixture the tight-binding ligand methotrexate was not observed in the electron-density maps (Zhao *et al.*, 2003 ▶). This was not considered to be an improvement on the results that we had previously obtained, so we elected to initiate crystallographic studies of *L. donovani* PTR1 (*Ld*PTR1), this also being the enzyme from the pathogen that causes the most serious form of leishmaniasis. There is a high level of conservation (91% sequence identity) between the *L. major* and *L. donovani* enzymes and homology modelling of the latter has suggested a close structural relationship in and around the active site (Kaur *et al.*, 2010 ▶).

## 2. Methods

### 2.1. Expression and purification

The gene encoding *Ld*PTR1 was cloned into the expression vector pET15b (Novagen) modified to encode a tobacco etch virus (TEV) protease-cleavable N-terminal hexahistidine tag. This plasmid was heat-shock transformed into *Escherichia coli* BL21 (DE3) GOLD cells (Stratagene) and selected on Luria–Bertani (LB) agar plates containing 50 mg l^−1^ carbenicillin. Cells were then cultured in LB medium containing the same antibiotic at 310 K with shaking. Gene expression was induced with isopropyl β-d-1-thiogalactopyranoside at a final concentration of 1 m*M* when the cells reached the mid-log phase of growth (optical density at 600 nm of 0.6–0.8). After incubation at room temperature for 16 h, the cells were harvested by centrifugation (4000*g*, 277 K, 30 min), washed with fresh medium and the centrifugation was repeated. Cell pellets were frozen at 253 K until required.

For purification, the frozen cell pellet from 1 l culture was thawed on ice and resuspended in lysis buffer (50 m*M* Tris–HCl pH 7.7, 200 m*M* KCl, 20 m*M* imidazole) supplemented with an EDTA-free protease-inhibitor cocktail tablet (Roche) and 100 µg DNAse I (Sigma–Aldrich). Cells were lysed using a French cell press and the lysate was clarified by centrifugation (37 000*g*, 277 K, 30 min). The supernatant was filtered (0.2 µm) and applied onto a 5 ml HisTrap metal-chelating column (GE Healthcare) preloaded with Ni^2+^ and equilibrated with lysis buffer. A linear imidazole gradient was applied and the protein eluted at a concentration of approximately 160 m*M* imidazole. Fractions containing *Ld*PTR1 were pooled and the histidine tag was cleaved by incubation with TEV protease at 303 K for 3 h. Imidazole was removed from the buffer by dialysis and cleavage continued at 277 K overnight before a second Ni^2+^-affinity step was performed, isolating the pure and cleaved *Ld*PTR1. The protein was concentrated to approximately 10 mg ml^−1^ using a centrifugal filter unit with a molecular-weight cutoff of 3500 Da (Millipore) and the buffer was exchanged to 20 m*M* Tris–HCl pH 7.7. The high level of purity (>95%) and the molecular weight of the protein were con­firmed by SDS–PAGE and matrix-assisted laser desorption/ionization time-of-flight mass spectrometry (data not shown). The yield of purified protein was relatively low at approximately 3 mg per litre of bacterial culture and we note that most of the material produced was insoluble.

### 2.2. Crystallization and data collection

**Figure 1:**
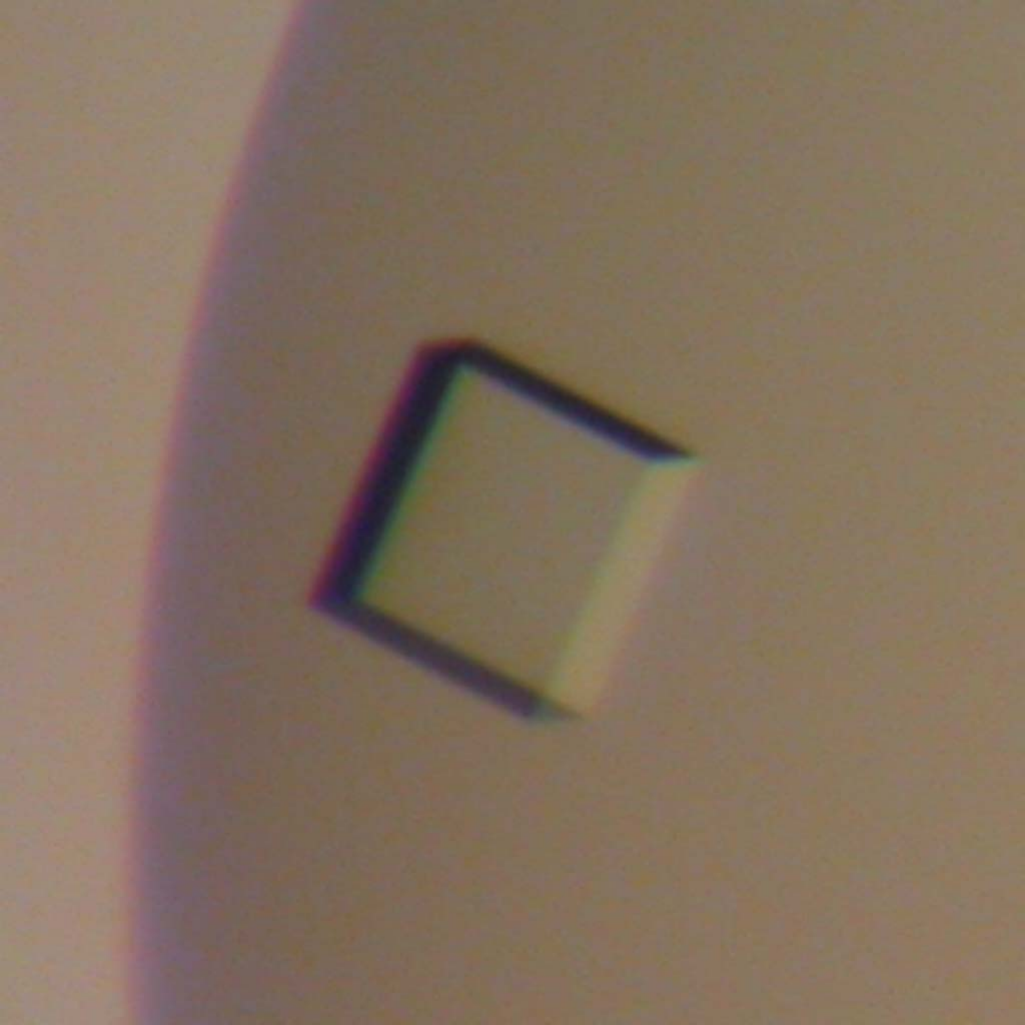
A crystal of *Ld*PTR1. The approximate dimensions of this sample were 0.1 × 0.1 × 0.03 mm.

Sitting-drop vapour diffusion was used to test a range of commercially available screens. Crystals were only obtained in the presence of both cofactor and an inhibitor and these were optimized in hanging drops. A solution of 5 mg ml^−1^ *Ld*PTR1, 1 m*M* NADP^+^, 20 m*M* dithiothreitol and 1 m*M* methotrexate was incubated on ice for 1 h before crystallization. The protein solution (1 µl) was mixed with reservoir solution (1 µl) and stored at room temperature. Small orthorhombic plates with approximate dimensions of 0.1 × 0.1 × <0.05 mm formed within days above reservoirs containing 0.1 *M* MES pH 6.5, 10%(*v*/*v*) dioxane and 1.6 *M* ammonium sulfate (Fig. 1 ▶).

Crystals were placed in a nylon loop and flash-cooled to 100 K in a stream of nitrogen gas after first being cryoprotected by passing them through a solution of 40% PEG 400. Initial in-house X-ray experiments using a Rigaku MicroMax-007 rotating-anode X-ray generator and an R-AXIS IV^++^ image-plate detector produced only smeared low-resolution diffraction images. The quality of the diffraction was improved by an annealing step in which the cryostream was manually diverted and reintroduced after 5–10 s (data not shown). Diffraction data were then collected on beamline I04 at the Diamond Light Source synchrotron.

### 2.3. Structure solution and refinement

The data were processed and scaled using *MOSFLM* (Leslie, 2006 ▶) and *SCALA* (Evans, 2006 ▶; Collaborative Computational Project, Number 4, 1994 ▶), respectively. The space group is *C*222_1_ and the unit-cell parameters are *a* = 107.51, *b* = 126.44, *c* = 87.51 Å. The Matthews coefficient (Matthews, 1968 ▶) of 2.5 Å^3^ Da^−1^, which corresponds to approximately 50% bulk solvent, suggested that two subunits occupy the asymmetric unit.

The *Ld*PTR1 structure was solved by molecular replacement using *Phaser* (McCoy *et al.*, 2007 ▶). The search model was a single *Lm*PTR1 subunit (PDB code 1e7w; Gourley *et al.*, 2001 ▶) in which divergent residues were truncated to C^β^ and all ligands were removed. Two subunits were positioned and rigid-body refinement and all subsequent refinements were carried out using *REFMAC*5 (Murshudov *et al.*, 1997 ▶). The graphics program *Coot* (Emsley & Cowtan, 2004 ▶) was used to inspect difference and electron-density maps for model fitting, solvent and ligand searching. Sulfate and water molecules were added after the protein model was completed and further rounds of refinement were performed. Noncrystallographic symmetry restraints were not employed during the analysis. Crystallographic statistics are shown in Table 1 ▶. Figures were prepared with *PyMOL* (DeLano, 2002 ▶).

## 3. Results and discussion

### 3.1. Overall structure

**Figure 2:**
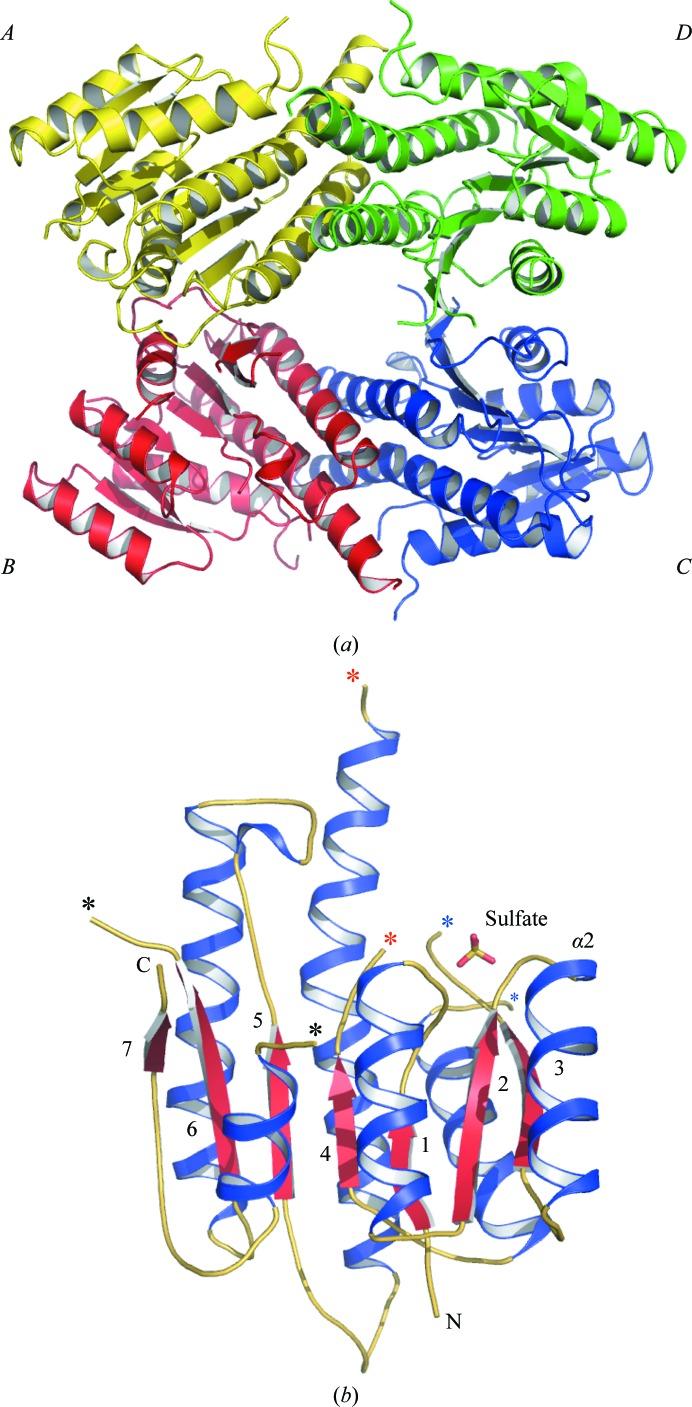
(*a*) Ribbon diagram of the *Ld*PTR1 tetramer. Subunits *A* and *B* constitute the asymmetric unit. (*b*) Ribbon diagram of an *Ld*PTR1 subunit. Seven red β-strands (numbered) are sandwiched between the blue α-helices; loop regions are coloured yellow. A sulfate is depicted as sticks in the active site with S coloured orange and O red; the N- and C-termini of the protein are labelled. Blue, red and black asterisks mark residues either side of the missing β3–α3, β4–α4 and β6–α6 loops, respectively.

PTR1 is a tetrameric enzyme and this crystal form of *Ld*PTR1 has two subunits (labelled *A* and *B*) in the asymmetric unit; a 2_1_ screw axis parallel to *c* generates the tetramer (Fig. 2 ▶ *a*). Each monomer is formed by a seven-stranded central β-sheet flanked by a set of three α-helices on either side (Fig. 2 ▶ *b*): a Rossmann-fold repeat (Gourley *et al.*, 2001 ▶). The structures of subunits *A* and *B* are highly conserved, with an r.m.s.d. of 0.51 Å when 212 C^α^ atoms are matched; therefore, unless otherwise stated all descriptions refer to subunit *A* of *Ld*PTR1.

### 3.2. A disordered active site

Sequence comparisons between *Ld*PTR1 and *Lm*PTR1 (data not shown) indicate that the residues involved in the construction of the active site and that are essential for catalysis in binding cofactor, substrates, products and inhibitors are strictly conserved and are also highly conserved in *Tb*PTR1 (Dawson *et al.*, 2006 ▶). However, the residues near the catalytic site were poorly ordered in contrast to the same regions of *Lm*PTR1 and *Tb*PTR1, with no electron density corresponding to NADP^+^ or methotrexate, ligands that were present in the crystallization conditions in a fivefold molar excess. The *K*_i_ for methotrexate inhibition of *Lm*PTR1 is 39 ± 19 n*M* and that with respect to *Tb*PTR1 is 152 ± 16 n*M* (Dawson *et al.*, 2006 ▶).

**Figure 3:**
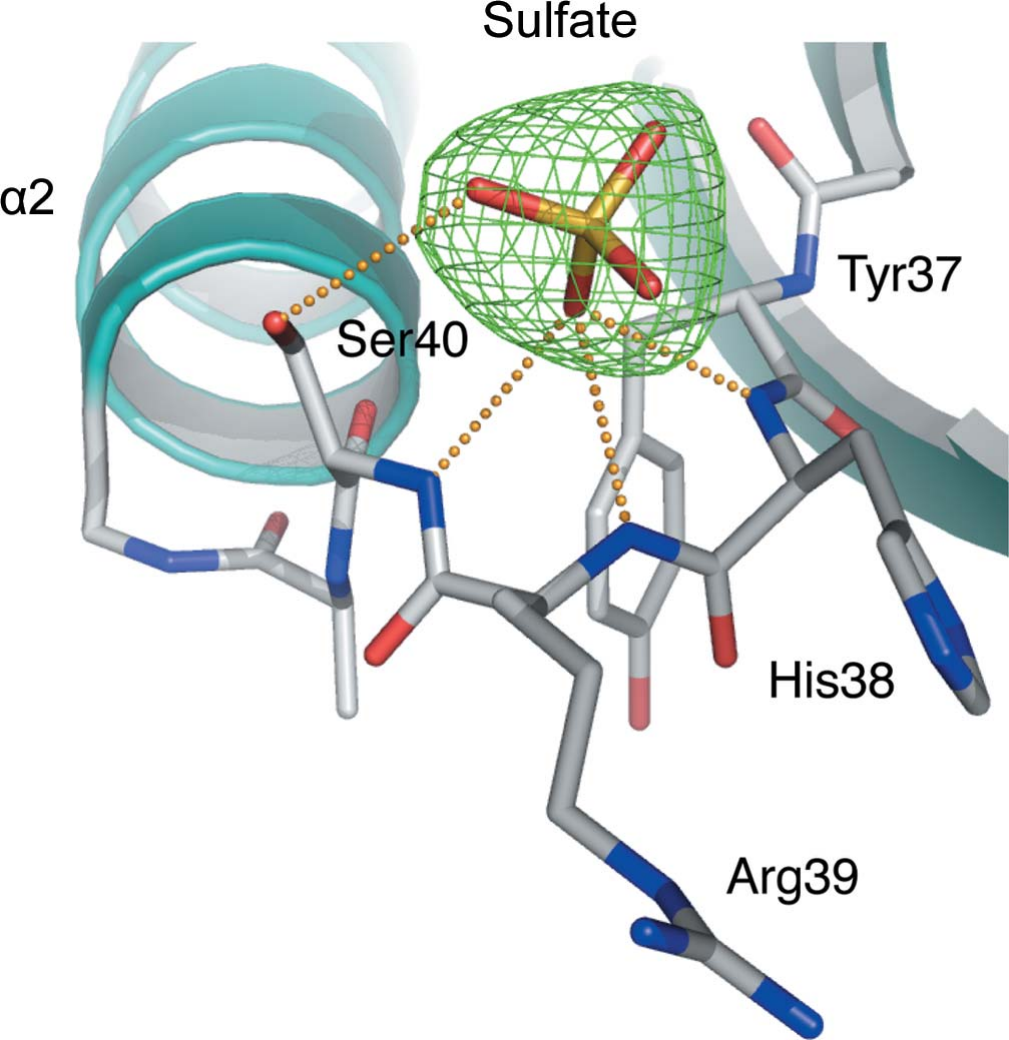
The *F*_o_ − *F*_c_ difference density OMIT map for sulfate bound in the *Ld*PTR1 NADP^+^-binding site (green mesh contoured at 3.5σ). The orange dotted lines represent potential hydrogen bonds formed between the ion and the enzyme. These interactions are in the range 2.7–3.1 Å.

Ammonium sulfate was the precipitant for crystal growth and a sulfate ion binds in a polar cavity formed by the β1–α1 turn and the loop between β2 and α2, accepting hydrogen bonds donated from three main-chain amides (His38, Arg39 and Ser40) and Ser40 OG (Fig. 3 ▶). This polar cavity is the binding site for the adenine 2′-­phosphate group of the cofactor (Gourley *et al.*, 2001 ▶; Dawson *et al.*, 2006 ▶).

**Figure 4:**
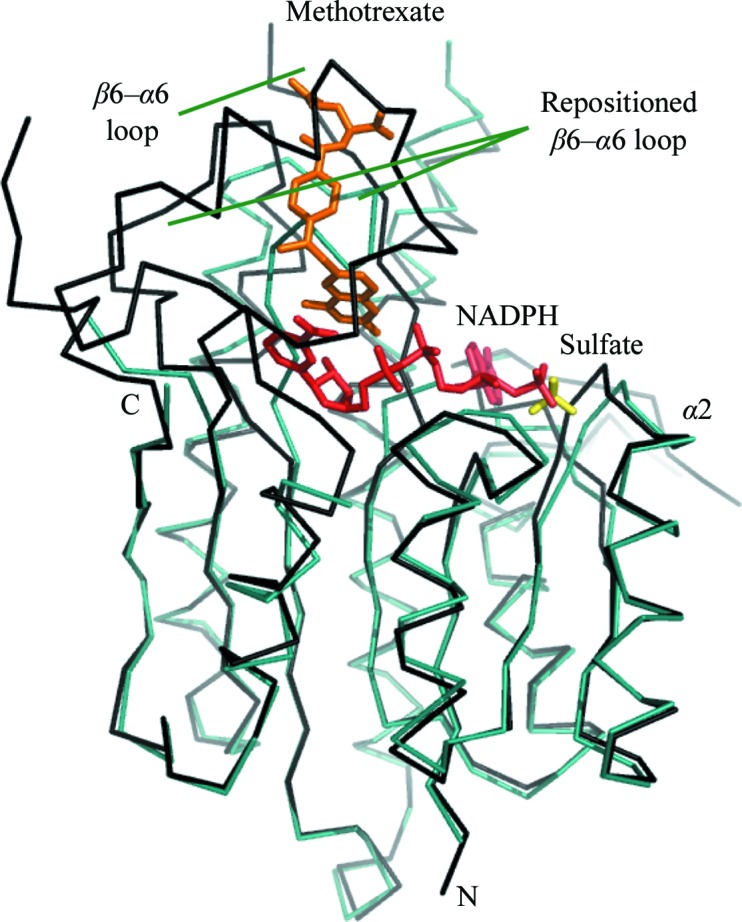
A C^α^ trace of one *Ld*PTR1 subunit (cyan) overlaid with an *Lm*PTR1 subunit (PDB code 1e7w; Gourley *et al.*, 2001 ▶; black). The sulfate ion bound to *Ld*PTR1 is shown as yellow sticks, while the NADPH and methotrexate binding to *Lm*PTR1 are depicted as red and orange sticks, respectively. The β6–α6 loop of the *Lm*PTR1 model is marked. This loop is absent from *Ld*PTR1. The β5–α5 substrate-binding loop adopts different positions in the two structures.

The core structure of the subunit is preserved between the *Ld*PTR1 and *Lm*PTR1 structures (Fig. 4 ▶) and an overlay of 202 C^α^ atoms common to both subunits resulted in an r.m.s.d. of 1.3 Å (calculated using *Coot*; Emsley & Cowtan, 2004 ▶). Owing to the absence of well defined electron density, the *Ld*PTR1 model contains fewer residues compared with those of *Lm*PTR1 or *Tb*PTR1. Missing segments include residues 70–80 (the loop linking β3 and α3), 112–132 (the β4–α4 loop) and 227–254 (the β6–α6 loop). The β3–α3 loop is also poorly ordered in *Lm*PTR1 (Schüttelkopf *et al.*, 2005 ▶). However, the β4–α4 loop is well ordered in *Lm*PTR1 but is missing in *Ld*PTR1. This means that in *Ld*PTR1, Phe113, which is a critical residue, is disordered. The phenylalanine, together with the nicotin­amide, forms π-stacking interactions that stabilize ligand binding in the catalytic site (Gourley *et al.*, 2001 ▶; Dawson *et al.*, 2006 ▶). The largest break in the electron density involves residues 227–254, which form what is termed the substrate-binding loop linking β6 to α6 (Tulloch *et al.*, 2010 ▶). Residues in this loop bind parts of folate sub­strates, products and some inhibitors. The absence of the β4–α4 loop vacates an area in the active-site cleft, allowing the β5–α5 loop of *Ld*PTR1 to adopt a different position to fill the gap. Here, the C^α^ atoms of several residues relocate by between 10 and 16 Å.

In *Ld*PTR1 Arg17 is disordered. This residue is important in binding the cofactor pyrophosphate (McLuskey *et al.*, 2004 ▶), which in turn interacts with and positions the nicotinamide. Asp181, Tyr194 and Lys198 are the key catalytic residues. Asp181 is located within the link between β5 and α5, but this loop has been built into weak electron density relative to the structure as a whole. The orientation of Tyr194 is similar to that observed in *Lm*PTR1. However, only the C^α^ atoms of Lys198 and its closest neighbours agree reasonably well with the identical *Lm*PTR1 residues, while the side chain extends into a position that is inappropriate to form the stabilizing interaction formed with the nicotinamide ribose in *Lm*PTR1 (Gourley *et al.*, 2001 ▶; data not shown).

PTR1 displays a sequential ordered mechanism, with first cofactor binding and then substrate; following reduction the product leaves, followed by oxidized cofactor (Luba *et al.*, 1998 ▶; Gourley *et al.*, 2001 ▶). Substrate or inhibitors can only bind after the binary protein–cofactor complex has formed. Since sulfate binding blocks the 2′-­phosphate-binding site, NADP^+^ is not present and this leads to disorder in the substrate-binding part of the active site.

## 4. Conclusions

The structure of apo *Ld*PTR1 has been solved to 2.5 Å resolution. Attempts to improve the *Ld*PTR1 crystal quality by attempting to crystallize the apo form and the binary complex with cofactor and by introducing other ligands in combination with oxidized and reduced cofactor failed. Even though we see no evidence for these ligands in the electron-density maps, the presence of both methotrexate and NADP^+^ was essential to obtain this crystal form. Despite their presence in the crystallization mixture, there was no electron density corresponding to these molecules. Instead, the high ammonium sulfate concentration in the crystallization conditions resulted in the replacement of an NADP^+^ phosphate by sulfate ions. The formation of the catalytic site of PTR1 is dependent on the presence of nicotinamide and in the absence of NADPH(^+^) this part of the active site is disordered. We conclude that owing to the disorder this crystal form is not suitable for characterizing the interactions of *Ld*PTR1 with inhibitors.

## Supplementary Material

PDB reference: pteridine reductase, 2xox

## Figures and Tables

**Table 1:** Data-collection and refinement statistics Values in parentheses are for the highest resolution shell.

Space group	*C*222_1_
Unit-cell parameters (Å)	*a* = 107.5, *b* = 126.4, *c* = 87.5
Resolution range (Å)	29.9–2.5 (2.6–2.5)
Wavelength (Å)	0.973
No. of measurements	144524 (21138)
No. of unique reflections	21004 (3022)
Multiplicity	6.9 (7.0)
Completeness (%)	99.9 (100.0)
Mean *I*/σ(*I*)	11.9 (3.8)
Wilson *B* factor (Å^2^)	46.9
*R*_merge_ † (%)	9.6 (42.4)
*R*_work_ ‡ (%)	22.7 (28.0)
*R*_free_ § (%)	28.5 (33.0)
R.m.s.d. bonds (Å)	0.019
R.m.s.d. bond angles (°)	1.802
Ramachandran analysis	
Favoured (%)	95.2
Allowed (%)	4.6
Outliers (%)	0.2
Protein residues (total)	432
Atoms (total)	3201
Overall *B* factor (Å^2^)	43.5
Additional groups	
Waters	
No.	24
Average *B* factor (Å^2^)	38.0
Sulfates	
No.	2
Average *B* factor (Å^2^)	53.4

† *R*_merge_ = 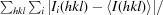 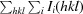, where *I_i_*(*hkl*) is the intensity of the *i*th measurement of reflection *hkl* and 〈*I*(*hkl*)〉 is the mean value of *I_i_*(*hkl*) for all *i* measurements.

‡ *R*_work_ = 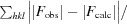 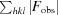, where *F*_obs_ is the observed structure-factor amplitude and *F*_calc_ is the structure-factor amplitude calculated from the model.

§ *R*_free_ is the same as *R*_work_ except calculated with a subset (5%) of data that were excluded from refinement calculations.

## References

[bb1] Cavazzuti, A., Paglietti, G., Hunter, W. N., Gamarro, F., Piras, S., Loriga, M., Allecca, S., Corona, P., McLuskey, K., Tulloch, L., Gibellini, F., Ferrari, S. & Costi, M. P. (2008). *Proc. Natl Acad. Sci. USA*, **105**, 1448–1453. 10.1073/pnas.0704384105 PMC2234164 18245389

[bb2] Collaborative Computational Project, Number 4 (1994). *Acta Cryst.* D**50**, 760–763.

[bb3] Croft, S. L., Sundar, S. & Fairlamb, A. H. (2006). *Clin. Microbiol. Rev.* **19**, 111–126. 10.1128/CMR.19.1.111-126.2006 PMC1360270 16418526

[bb4] Cunningham, M. L., Titus, R. G., Turco, S. J. & Beverley, S. M. (2001). *Science*, **292**, 285–287. 10.1126/science.1057740 11303103

[bb5] Dawson, A., Gibellini, F., Sienkiewicz, N., Tulloch, L. B., Fyfe, P. K., McLuskey, K., Fairlamb, A. H. & Hunter, W. N. (2006). *Mol. Microbiol.* **61**, 1457–1468. 10.1111/j.1365-2958.2006.05332.x PMC1618733 16968221

[bb6] Dawson, A., Tulloch, L. B., Barrack, K. L. & Hunter, W. N. (2010). *Acta Cryst.* D**66**, 1334–1340. 10.1107/S0907444910040886 PMC3655514 21123874

[bb7] DeLano, W. L. (2002). *PyMOL.* http://www.pymol.org.

[bb8] Desjeux, P. (2004). *Comp. Immunol. Microbiol. Infect. Dis.* **27**, 305–318. 10.1016/j.cimid.2004.03.004 15225981

[bb9] Emsley, P. & Cowtan, K. (2004). *Acta Cryst.* D**60**, 2126–2132. 10.1107/S0907444904019158 15572765

[bb10] Evans, P. (2006). *Acta Cryst.* D**62**, 72–82. 10.1107/S0907444905036693 16369096

[bb11] Gibson, C. L., Huggan, J. K., Kennedy, A., Kiefer, L., Lee, J. H., Suckling, C. J., Clements, C., Harvey, A. L., Hunter, W. N. & Tulloch, L. B. (2009). *Org. Biomol. Chem.* **7**, 1829–1842. 10.1039/b818339b 19590778

[bb12] Gourley, D. G., Schüttelkopf, A., Leonard, G., Luba, J., Hardy, L., Beverley, S. & Hunter, W. N. (2001). *Nature Struct. Biol.* **8**, 521–525. 10.1038/88584 11373620

[bb13] Herwaldt, B. L. (1999). *Lancet*, **354**, 1191–1199. 10.1016/S0140-6736(98)10178-2 10513726

[bb14] Hunter, W. N. (2009). *J. Biol. Chem.* **284**, 11749–11753. 10.1074/jbc.R800072200 PMC2673241 19103598

[bb15] Leslie, A. G. W. (2006). *Acta Cryst.* D**62**, 48–57. 10.1107/S0907444905039107 16369093

[bb16] Luba, J., Nare, B., Liang, P.-H., Anderson, K. S., Beverley, S. M. & Hardy, L. W. (1998). *Biochemistry*, **37**, 4093–4104. 10.1021/bi972693a 9521731

[bb17] Kaur, J., Sundar, S. & Singh, N. (2010). *J. Antimicrob. Chemother.* **65**, 1742–1748. 10.1093/jac/dkq189 20519355

[bb18] Maltezou, H. C. (2010). *J. Biomed. Biotechnol.* **2010**, 617521. 10.1155/2010/617521 PMC2771279 19888437

[bb19] Matthews, B. W. (1968). *J. Mol. Biol.* **33**, 491–497. 10.1016/0022-2836(68)90205-2 5700707

[bb20] McCoy, A. J., Grosse-Kunstleve, R. W., Adams, P. D., Winn, M. D., Storoni, L. C. & Read, R. J. (2007). *J. Appl. Cryst.* **40**, 658–674. 10.1107/S0021889807021206 PMC2483472 19461840

[bb21] McLuskey, K., Gibellini, F., Carvalho, P., Avery, M. A. & Hunter, W. N. (2004). *Acta Cryst.* D**60**, 1780–1785. 10.1107/S0907444904018955 15388924

[bb22] Moreira, W., Leblanc, E. & Ouellette, M. (2009). *Free Radic. Biol. Med.* **46**, 367–375. 10.1016/j.freeradbiomed.2008.10.034 19022374

[bb23] Mpamhanga, C. P., Spinks, D., Tulloch, L. B., Shanks, E. J., Robinson, D. A., Collie, I. T., Fairlamb, A. H., Wyatt, P. G., Frearson, J. A., Hunter, W. N., Gilbert, I. H. & Brenk, R. (2009). *J. Med. Chem.* **52**, 4454–4465. 10.1021/jm900414x PMC2966039 19527033

[bb24] Murshudov, G. N., Vagin, A. A. & Dodson, E. J. (1997). *Acta Cryst.* D**53**, 240–255. 10.1107/S0907444996012255 15299926

[bb25] Nare, B., Hardy, L. W. & Beverley, S. M. (1997). *J. Biol. Chem.* **272**, 13883–13891. 10.1074/jbc.272.21.13883 9153248

[bb26] Nare, B., Luba, J., Hardy, L. W. & Beverley, S. (1997). *Parasitology*, **114**, 101–110. 9309772

[bb27] Reithinger, R., Dujardin, J. C., Louzir, H., Pirmez, C., Alexander, B. & Brooker, S. (2007). *Lancet Infect. Dis.* **7**, 581–596. 10.1016/S1473-3099(07)70209-8 17714672

[bb28] Schormann, N., Pal, B., Senkovich, O., Carson, M., Howard, A., Smith, C., DeLucas, L. & Chattopadhyay, D. (2005). *J. Struct. Biol.* **152**, 64–75. 10.1016/j.jsb.2005.07.008 16168672

[bb32] Schüttelkopf, A. W., Hardy, L. W., Beverley, S. M. & Hunter, W. N. (2005). *J. Mol. Biol.* **352**, 105–116. 10.1016/j.jmb.2005.06.076 16055151

[bb29] Tulloch, L. B., Martini, V. P., Iulek, J., Huggan, J. K., Lee, J. H., Gibson, C. L., Smith, T. K., Suckling, C. J. & Hunter, W. N. (2010). *J. Med. Chem.* **53**, 221–229. 10.1021/jm901059x PMC2804273 19916554

[bb30] World Health Organization. (2007). *Global Plan to Combat Neglected Tropical Diseases 2008–2015.* http://whqlibdoc.who.int/hq/2007/WHO_CDS_NTD_2007.3_eng.pdf.

[bb31] Zhao, H., Bray, T., Ouellette, M., Zhao, M., Ferre, R. A., Matthews, D., Whiteley, J. M. & Varughese, K. I. (2003). *Acta Cryst.* D**59**, 1539–1544. 10.1107/s0907444903013131 12925782

